# Unraveling the mechanistic features of RNA polymerase II termination by the 5′-3′ exoribonuclease Rat1

**DOI:** 10.1093/nar/gkv133

**Published:** 2015-02-26

**Authors:** Jieun Park, Myungjin Kang, Minkyu Kim

**Affiliations:** Center for RNA Research, Institute for Basic Science and Department of Biophysics and Chemical Biology, Seoul National University, 1 Gwanak-Ro, Gwanakgu, Seoul, 151-742, South Korea

## Abstract

Within a complex with Rai1, the 5′-3′ exoribonuclease Rat1 promotes termination of RNA polymerase II (RNAPII) on protein-coding genes, but its underlying molecular mechanism is still poorly understood. Using *in vitro* transcription termination assays, we have found that RNAPII is prone to more effective termination by Rat1/Rai1 when its catalytic site is disrupted due to NTP misincorporation, implying that paused RNAPII, which is often found *in vivo* near termination sites, could adopt a similar configuration to Rat1/Rai1 and trigger termination. Intriguingly, yeast Rat1/Rai1 does not terminate *Escherichia coli* RNAP, implying that a specific interaction between Rat1/Rai1 and RNAPII may be required for termination. Furthermore, the efficiency of termination increases as the RNA transcript undergoing degradation by Rat1 gets longer, which suggests that Rat1 may generate a driving force for dissociating RNAPII from the template while degrading the nascent transcripts to catch up to the polymerase. These results indicate that multiple mechanistic features contribute to Rat1-mediated termination of RNAPII.

## INTRODUCTION

Three classes of RNA polymerases (RNAPs) exist in eukaryotes: RNAPI transcribes rRNAs, RNAPII transcribes mRNAs and a majority of non-coding RNAs (sn/snoRNA, miRNA, CUTs, SUTs), and RNAPIII transcribes 5S rRNA and tRNAs. Accurate transcription termination is important because early or late termination may disrupt normal gene regulation and produce abnormal RNAs, which may be harmful to cellular fitness.

In *Saccharomyces cerevisiae*, there are at least two distinct pathways for RNAPII termination (1). One pathway involves an Nrd1/Nab3/Sen1 complex that terminates non-coding RNA transcription and is independent of cleavage of nascent RNA transcripts (2,3). In contrast, the other termination pathway for most protein-coding genes requires cleavage of nascent transcripts by cleavage/polyadenylation factors and RNA degradation from the newly formed 5′ phosphorylated end by 5′-3′ exoribonuclease Rat1 promotes RNAPII termination (4,5).

The yeast Rat1 is an essential nuclear protein and is evolutionarily well conserved from yeast to human (Xrn2 in human) (5–7). Rat1 forms a complex with Rai1 that stabilizes Rat1 and helps target 5′ monophosphate RNA by its pyrophosphohydrolase activity (8). Rtt103 is another interacting protein that has a CID (RNAPII C-terminal domain-interacting domain) that might facilitate the access of Rat1 to RNAPII by interacting with the connective tissue disease (CTD), which is phosphorylated at serine 2 (4,9). A study involving a scaffold transcription elongation complex insisted that Rat1/Rai1 itself is not sufficient to terminate RNAPII *in vitro* (10). However, another *in vitro* study using a promoter-driven elongation complex showed that Rat1/Rai1 released stalled RNAPII in the absence of other factors (9). Thus, the factors required by Rat1 to promote termination and the mechanism of RNAPII dissociation still remain obscure.

Because RNAP moves by Brownian motion rather than by adenosine triphosphate (ATP)-driven power strokes (11–13), frequent pausing, backtracking and transcriptional arrest are commonly observed. A major factor responsible for RNAPII pausing could be nucleoside 5′-triphosphate (NTP)-misincorporation. According to recent work on RNAPs, a non-cognate NTP complementary to the *n* + 1 template DNA base (*n* + 1 NTP) can be incorporated through template misalignment, leading to a temporary movement of a DNA base to an extrahelical position (14,15). If nucleotides are mismatched, the RNA transcript can be extended after realignment of template DNA. This misalignment mechanism seems to be universal for all DNA-dependent RNAPs, including bacterial RNAP (14,15). The average frequency of NTP misincorporation remains relatively low for eukaryotic RNAPII (∼10^−5^) (16) because Rpb9 and TFIIS stimulate the proofreading cleavage activity of RNAPII (17–20). Supporting these results, deletion of the *RPB9* or TFIIS (*DST1*) gene significantly decreases the transcriptional fidelity of yeast RNAPII (21). NTP misincorporation would arrest the elongation complex and/or induce transient catalytic inactivation of the elongation complex (22,23). In addition to template misalignment-induced pausing, sequence-specific pausing occurs in various RNAPs. In yeast RNAPII, a mismatched DNA/RNA hybrid T·U wobble base pair induces disruption of the catalytic site, resulting in RNAPII pausing and backtracking (23). In addition, bacterial and *Thermus thermophilus* RNAPs also show sequence-specific pausing, depending on the NTP that is entering the active site (24,25). Several studies have reported that RNAPII pausing is linked to and stimulates termination by Rat1/Xrn2 (26,27), but other than its interaction with a poly(A) signal, the mechanism by which RNAPII pausing contributes to Rat1-mediated termination remains unknown.

In this study using *in vitro* assays, we show that when an NTP misincorporates, RNAPII becomes catalytically disrupted and is more efficiently terminated by Rat1/Rai1. These results suggest that the pausing of RNAPII, which often occurs *in vivo* near the termination site, might generate a similar configuration that facilitates termination by Rat1/Rai1. Although NTP misincorporation also catalytically disrupts *Escherichia coli* RNAP, Rat1/Rai1 does not terminate it, indicating that a specific interaction between Rat1/Rai1 and RNAPII may also be important to induce termination. Additionally, we found that RNAPII termination efficiency depends on the length of RNA transcript undergoing degradation by Rat1, which suggests that Rat1 may generate a driving force to dislodge RNAPII while degrading the nascent transcripts to catch up to the polymerase.

## MATERIALS AND METHODS

### Strains and plasmids construction

The genes encoding for *S. cerevisiae* Rat1, Rai1 and Rtt103 were cloned into pET21b (Novagen, Germany) tagged with hexahistidine (6xHis) (gifts from P. Cramer Ludwig-Maximilians Universität, Munich, Germany). Human Xrn2 (hXrn2) was cloned from cDNA (Harvard DNA Resources) into pET21b tagged with 6xHis. Catalytic mutants of rat1 (E203A, D233A, D235A) and rai1 (E221A, D223A) were prepared by site-directed mutagenesis. Xrn1 was purchased from NEB (cat# M0338S).

The rat1 E203A/D233A/D235A (rat1EDD) mutant was generated by polymerase chain reaction mutagenesis and cloned into either pET21b or pRS41H. Rtt103 was cloned into pRS415 or pRS423 and transformed into a yeast strain (rat1Δ::KanMX/pAJ202-Rat1/pRS41H-rat1EDD) to test whether extra copies of the Rtt103 gene could rescue the rat1EDD lethal phenotype when wild-type Rat1 was shuffled out by 5-Fluoroorotic Acid (5-FOA) selection.

### Protein expression and purification

The 6xHis-tagged recombinant proteins of Rat1, Rai1, Rtt103 and TFIIS were over-expressed in a BL21 CodonPlus (DE3) RIL (Stratagene) strain via IPTG induction (0.25 mM IPTG) at 25°C for 6 h.

Cells expressing Rat1/Rai1 or Rat1 were lysed by sonication in freezing buffer A (50 mM Tris–HCl, pH 7.9 at 24°C, 150 mM NaCl, 10% glycerol, 10 mM β-ME, 1.8 μM leupeptin, 5.46 μM pepstatin A, 6.33 mM benzamidine, 37.5 μg bestatin, 3 mM Phenylmethylsulfonyl fluoride (PMSF)). The lysates were cleared by centrifugation and sequentially applied to an Ni-NTA agarose column (Qiagen), a HiTrap Heparin HP affinity column (5 ml, GE Healthcare), and a MonoQ 10/100 GL anion exchange column (GE Healthcare) and underwent Superose6 10/300 GL size-exclusion chromatography. The purified proteins were eluted in final buffer (25 mM Tris–HCl, pH 7.9 at 24°C, 100 mM NaCl, 1 mM MgCl_2_, 1 mM Dithiothreitol (DTT), 10% (v/v) glycerol), quick frozen in liquid nitrogen and stored at −80°C. The Rat1 variants were purified by the same procedure. For Rtt103, cells were lysed by sonication in freezing buffer B (50 mM Tris–HCl, pH 7.3 at 24°C, 150 mM NaCl, 10% glycerol, 10 mM β-ME, 1.8 μM leupeptin, 5.46 μM pepstatin A, 6.33 mM benzamidine, 37.5 μg bestatin, 3 mM PMSF) and the cell lysate was sequentially applied to Ni-NTA, MonoQ and Superose 6 columns. The purified protein was eluted in final buffer B (25 mM Tris–HCl, pH 6.8 at 24°C, 125 mM NaCl, 1.25 mM DTT, 10% (v/v) glycerol). For TFIIS, purification procedure was performed as described previously (28).

The Rat1/Rai1/Rtt103 and rat1EDD/Rai1/Rtt103 complexes were purified using the same procedures but with slightly different buffer conditions. In the affinity and ion-exchange chromatography steps, Tris–HCl, pH 7.6 at 24°C was used. In the Superpose 6 size-exclusion chromatography step, protein complexes were eluted with low-salt storage buffer (25 mM Tris–HCl, pH 7.6 at 24°C, 80 mM NaCl, 1 mM DTT, 1 mM MgCl_2_, 10% (v/v) glycerol).

For *S. cerevisiae* RNAPII purification, the BJ5464 Rpb3 His-Bio strain (a gift from P. Cramer) was fermented and purified as described previously (29). The Rpb4/7 subunit was over-expressed in a BL21 CodonPlus (DE3) RIL (Stratagene) strain via IPTG induction (0.25 mM IPTG) at 25°C for 6 h. It was purified as described elsewhere (30).

### *In vitro* transcription termination assay

Transcription-competent elongation complexes were assembled as previously described (10). The template/non-template DNAs and RNAs used to assemble an elongation complex (EC) are listed in Supplementary Table S1. Briefly, 3 pmol of RNAPII was incubated with a two-fold molar excess of DNA/RNA hybrid, a four-fold molar excess of 5′-biotinylated non-template DNA and a five-fold molar excess of Rbp4/7 to form the EC. Streptavidin-coated magnetic beads (Dynabeads MyOne streptavidin T1, Invitrogen) were pre-blocked O/N with blocking buffer (50 mM Tris–HCl, pH 8.0 at 25°C, 150 mM NaCl, 2 mM ethylenediaminetetraacetic acid (EDTA), 0.1% (w/v) Triton X-100, 5% (w/v) glycerol, 0.5% (w/v) bovine serum albumin, 0.2 mg/ml insulin, 0.1 mg/ml heparin, 0.5 mM DTT) to prevent the non-specific binding of ECs. After the ECs were bound to the beads, the 3′-ends of the RNA molecules were labeled with [α-^32^P] uridine 5′-triphosphate (UTP) by RNAPII.

For RNA digestion, 6 pmol of Rat1/Rai1 was added to the ECs and the samples were incubated for 1 h at 30°C in the presence or absence of each NTP. After the reaction was complete, the nuclease and NTP were removed with washing buffer (20 mM Tris–HCl, pH 8.0 at 30°C, 500 mM NaCl, 2 mM MgCl_2_, 1 mM DTT) and the EC bound beads were resuspended in reaction buffer (20 mM Tris–HCl, pH 8.0 at 30°C, 150 mM NaCl, 2 mM MgCl_2_, 1 mM DTT). A mixture of four NTPs was added to EC and the samples were incubated for 30 min at 28°C to allow RNAPII elongation. The reactions were stopped and RNA samples were analyzed by 7 M Urea-polyacrylamide gel electrophoresis. The radioactively labeled RNA was detected by phosphorimaging (BAS-5000, Fujifilm).

### ATPase activity assay

An adenosine triphosphatase (ATPase) activity assay was performed as previously described (31). Each 20-μl reaction contained 0.66 pmol of [γ-^32^P] ATP, 6 pmol of nucleases, 5.6 pmol of DNA or RNA and 0.2 mM of MgCl_2_. The reaction mixture was incubated for 45 min at 37°C and terminated by the addition of EDTA to 0.5 mM and cold ATP to 0.6 mM. Approximately 2 μl from each reaction was spotted onto a thin-layer chromatography Polyethyleneimine (PEI) plate (Merck) and developed in 0.6 M KH_2_PO_4_ (pH 3.4). The extent of ATP hydrolysis was detected using phosphorimaging.

## RESULTS

### Rat1/Rai1 efficiently terminates RNAPII *in vitro* in the presence of ATP

**Figure 1.**
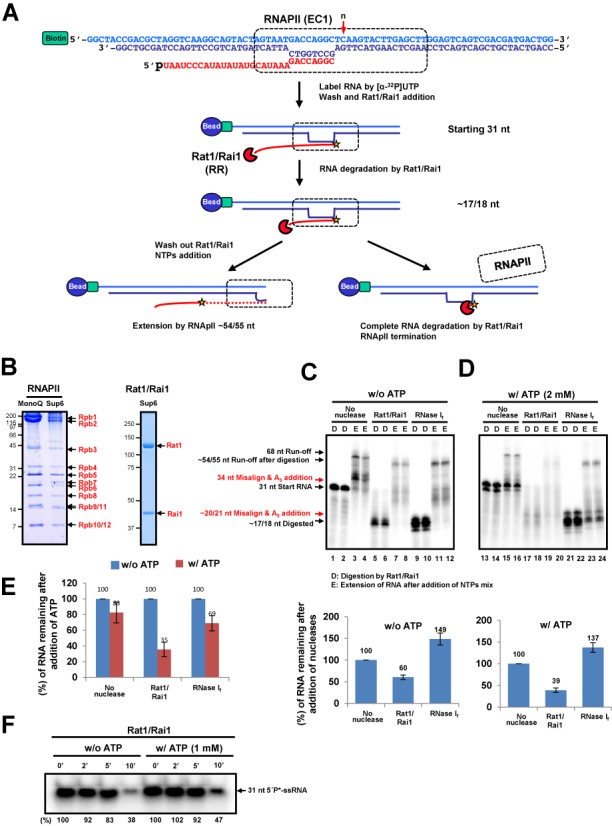
Rat1/Rai1 terminates RNAPII *in vitro* in the presence of ATP. (**A**) *In vitro* transcription termination assay scheme. The EC was assembled with double-stranded DNA (EC1), 5′ phosphorylated RNA and purified RNAPII and was subsequently coupled to magnetic beads. The 3′-end of RNA was radioactively labeled by RNAPII. Rat1/Rai1 digested RNA from 5′ to 3′ up to the surface of RNAPII. If Rat1/Rai1 failed to terminate RNAPII, the polymerase would elongate further using NTPs. However, if Rat1/Rai1 terminated RNAPII, the remaining RNA, which was protected by RNAPII, would be completely degraded by Rat1/Rai1. The red arrow specifies the position (*n*) of the first incoming NTP. (**B**) Purified RNAPII complex and Rat1/Rai1 on gels stained with Coomassie. (**C**) Representative gel image of the *in vitro* transcription termination assay with Rat1/Rai1 treatment in the absence of ATP. Rat1/Rai1 did not terminate RNAPII by itself. Black arrows indicate the RNAs predicted in (A). Red arrows show ATP-misincorporated RNAs. (**D**) digestion by Rat1/Rai1. (**E**) Extension of RNA after addition of NTPs mixture. Quantification of the remaining RNA compared with the control lacking nuclease (set to 100%) is shown below. (D) Rat1/Rai1 terminates RNAPII efficiently in the presence of 2 mM ATP. (E) Quantification of the remaining RNAs after ATP treatment compared with the no ATP control (set to 100%). (**F**) Time course showing the degradation of 31-nt single strand RNA substrate by Rat1/Rai1 in the absence or presence of 1 mM ATP. The amount of remaining RNA is presented as a percentage of that from the 0 min reaction after Rat1/Rai1 treatment. Asterisk represents radioactive labeling at 5′end of 31-nt RNA.

To unravel the mechanism of RNAPII termination, we adopted an *in vitro* transcription termination assay that was previously developed by Cramer *et* *al*. (Figure 1A) (10). This simplified system mimics an EC and consists of double-stranded DNA, 31 nt 5′-phosphorylated RNA and purified RNAPII. The ECs were immobilized to streptavidin-coated magnetic beads with the use of biotin at the 5′-end of the non-template DNA strand. The 3′-end of the RNA was labeled via [α-^32^P] UTP incorporation by RNAPII. After washing out unincorporated [α-^32^P] UTP, Rat1/Rai1 was added to the ECs in the presence or absence of other factors and RNAPII termination was monitored. In this setup, Rat1/Rai1 initially degraded the RNA up to the surface of RNAPII (∼17/18 nt). Once it had interacted with Rat1/Rai1, if RNAPII was terminated, the RNA would no longer be protected by the polymerase and would be degraded by Rat1. In contrast, if RNAPII was not terminated, the template-bound polymerase would continue to elongate in the presence of NTPs, generating an ∼54/55 nt run-off transcript (Figure 1A). The RNAPII and Rat1/Rai1 were expressed and purified from *S. cerevisiae* and *E. coli*, respectively (Figure 1B).

When Rat1/Rai1 was added to the ECs, the vast majority of RNAs were degraded up to ∼17/18 nt (Figure 1C, lanes 5 and 6) but subsequently extended to ∼54/55 nt by RNAPII (Figure 1C, lanes 7 and 8). RNA signals from the Rat1/Rai1-treated ECs were ∼60% of the control (no nuclease or NN) (compare the RNA bands from D lanes in Figure 1C), indicating that Rat1/Rai1 alone terminated RNAPII by an unknown mechanism, but that termination was very inefficient, as previously reported (10) (Figure 1C, lower panel). However, we surprisingly found that the remaining RNA level was dramatically decreased by Rat1/Rai1 when ATP (2 mM) was added (Figure 1D, lanes 16 and 17), indicating that ATP significantly enhanced Rat1/Rai1-mediated RNAPII termination. Titration experiments detected a noticeable improvement of termination at an ATP concentration as low as 0.5 mM (data not shown). These results show that Rat1/Rai1 is sufficient to terminate RNAPII *in vitro* in the presence of ATP. Notably, the addition of ATP with RNase I_f_ did not lead to a similar decrease in the amount of RNA, indicating that this ATP-dependent effect seems to be unique to Rat1/Rai1 in triggering RNAPII termination (Figure 1E).

To test whether the enhanced RNAPII termination by Rat1/Rai1 is due to the stimulation of exonuclease activity by ATP addition, we analyzed the rate of exoribonuclease activity of Rat1/Rai1 in the absence and presence of ATP. The remaining 5′end radioactively labeled RNA amount after the treatment of Rat1/Rai1 was measured along the indicated time course in the absence or presence of ATP (Figure 1F). As expected, ATP addition did not stimulate the processivity of Rat1. Rather, it slightly reduced the exonuclease activity because 1 mM of ATP was sufficient to chelate the Mg^2+^ ion necessary for the nuclease activity of Rat1, suggesting that ATP affects Rat1-mediated termination in a somewhat different manner.

### Rat1/Rai1 does not have ATPase activity

**Figure 2.**
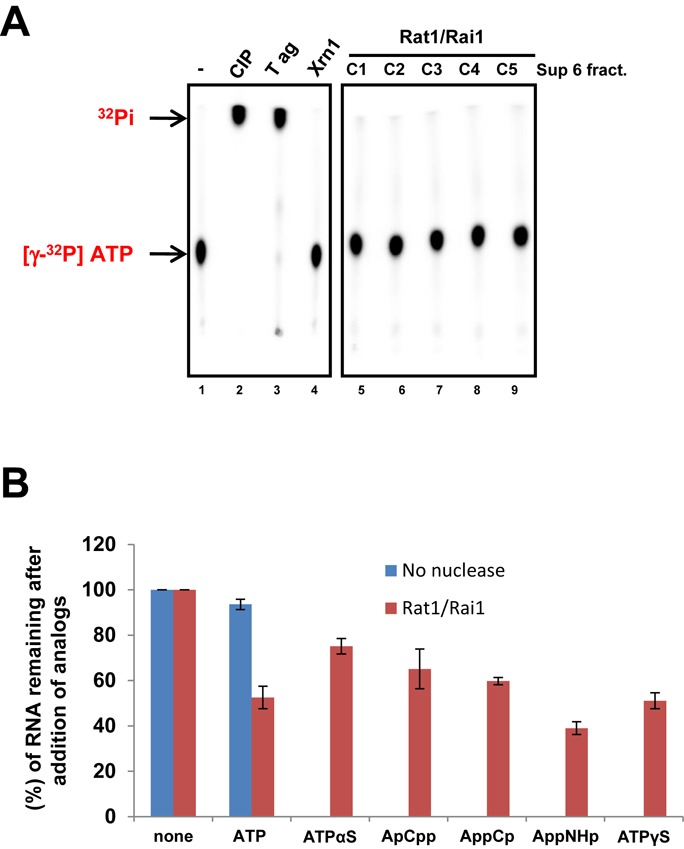
Rat1/Rai1 does not have ATPase activity. (**A**) ATPase activity assay of Rat1/Rai1. Calf intestinal phosphatase (CIP), SV40 T antigen (T ag) and Xrn1 were used as controls. Highly purified Rat1/Rai1 fractions from size-exclusion chromatography (Superose 6) show no ATPase activity. (**B**) Quantification of the remaining RNAs from the *in vitro* transcription termination assay with various non-hydrolyzable ATP analogs.

We first postulated that ATP-dependent RNAPII termination might occur via ATP hydrolysis by Rat1/Rai1. Because the 5′-pyrophosphohydrolase activity of Rai1 is limited only to RNA substrates (32), we tested whether Rat1 had ATP hydrolyzing activity. Several fractions of highly purified Rat1/Rai1 from size-exclusion chromatography (Superose 6) were incubated with [γ-^32^P] ATP and the reaction mixtures were resolved by thin-layer chromatography to detect hydrolyzed inorganic phosphate. Although calf intestinal phosphatase and SV40 T antigen (T ag) readily hydrolyzed ATP, Rat1/Rai1 did not show ATPase activity (Figure 2A, lanes 5–9). Similarly, Xrn1 did not have ATPase activity (Figure 2A, lane 4), as predicted from the amino acid sequences of XRN family proteins.

When non-hydrolyzing ATP analogs were used in the termination assay, the levels of RNAs (∼17/18 nt) that remained were higher than those observed in the ATP-treated controls (Figure 2B), indicating that RNAPII termination by Rat1/Rai1 was somewhat reduced by these non-hydrolyzing analogs. These results suggest that ATP hydrolysis may be crucial to promote RNAPII termination but is not driven by Rat1/Rai1.

### NTP misincorporation induces RNAPII pausing and enhances termination by Rat1/Rai1

**Figure 3.**
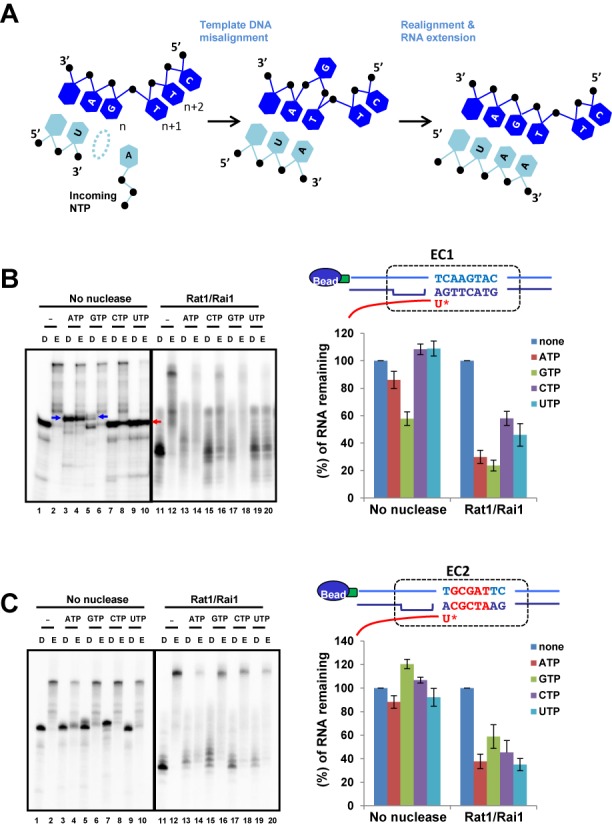
NTP misincorporation induces RNAPII pausing and enhances termination by Rat1/Rai1. (**A**) Schematic detail for NTP misincorporation via template misalignment. The RNA strand is shown in sky blue and the DNA strand is in dark blue. (**B**) EC1 scaffold tested. DNA sequences are shown on top of the quantification graph. An asterisk specifies the incorporation site of an incoming NTP (*n* position). In a control group lacking nuclease, the addition of non-cognate ATP or GTP generated misincorporated RNA bands via template misalignment (blue arrows, ∼34/35 nt), whereas cognate CTP does not. Notably, Rat1/Rai1 more efficiently terminates RNAPII when non-cognate NTP (ATP or GTP) rather than cognate CTP was added. Addition of UTP to EC1 induces strong pausing of RNAPII that does not support further elongation (red arrow), which results in slight inhibition of termination by Rat1/Rai1. (**B**) EC2 scaffold tested. Different sequences are shown in red. Rat1/Rai1 more effectively terminates RNAPII when a non-cognate NTP (ATP, CTP or UTP), rather than cognate GTP, was added.

Intriguingly, we observed an additional RNA band when ATP was added to the assay: ∼34 nt in the control without nuclease and ∼20/21 nt in the RNase I-treated group (Figure 1D, lanes 1–4 and 9–12). These RNAs seem to be generated by ATP misincorporation via template misalignment (14,15) because ATP is complementary to thymine at the *n* + 1 and *n* + 2 positions of the template DNA strand (Figure 3A). When ATP misincorporates into RNA transcripts, we observed that the elongation of RNAPII was significantly blocked (Figure 1D), presumably due to the disruption and/or rearrangement of the RNAPII active center (23,33). We hypothesized that RNAPII with a disrupted active center might be more effectively terminated by Rat1/Rai1. To investigate whether NTP misincorporation could induce RNAPII pausing and enhance subsequent termination by Rat1/Rai1, other NTPs were separately added to RNAPII assembled in the same EC1 scaffold (Figure 3B). In fact, non-cognate Guanosine 5′-triphosphate (GTP) also resulted in longer (∼34/35 nt) RNA transcripts via template misalignment and less RNAPII elongation (Figure 3B, lanes 5 and 6). When GTP was co-treated with Rat1/Rai1, it caused RNAPII termination as efficiently as ATP (Figure 3B; compare lanes 13 and 17). Another non-cognate UTP produced ∼32 nt RNA transcripts and strongly blocked RNAPII elongation (Figure 3B, lanes 9 and 10), most likely because UTP misincorporation resulted in a UU pause sequence at the 3′-end of the RNA, which adopts a frayed position in the pore below the active center (33). Upon adding Rat1/Rai1, UTP induced RNAPII termination, albeit far less efficiently than ATP did (Figure 3B, lanes 19 and 20). However, the cognate CTP did not cause significant RNAPII pausing nor termination compared with non-cognate ATP and GTP (Figure 3B, compare lanes 7 and 8 with lanes 15 and 16). To further verify this NTP misincorporation effect, a different DNA sequence template (EC2 scaffold) was tested with the assay (Figure 3C). Again, non-cognate NTPs (ATP, UTP and CTP) led to RNAPII termination much more efficiently than cognate GTP did (Figure 3C, lanes 13, 17, 19 and 15), confirming that NTP misincorporation indeed affected RNAPII termination by Rat1/Rai1. Although the incoming UTP is non-cognate to both EC scaffolds tested and generates a mismatched UU sequence, its effect on RNAPII pausing and termination by Rat1/Rai1 varied significantly at each EC scaffold (Figure 3B and C), suggesting that the sequence context may also affect the efficiencies of NTP misincorporation, RNAPII pausing and the subsequent termination by Rat1/Rai1.

### Other 5′-3′ exoribonucleases and *E. coli* RNAP

**Figure 4.**
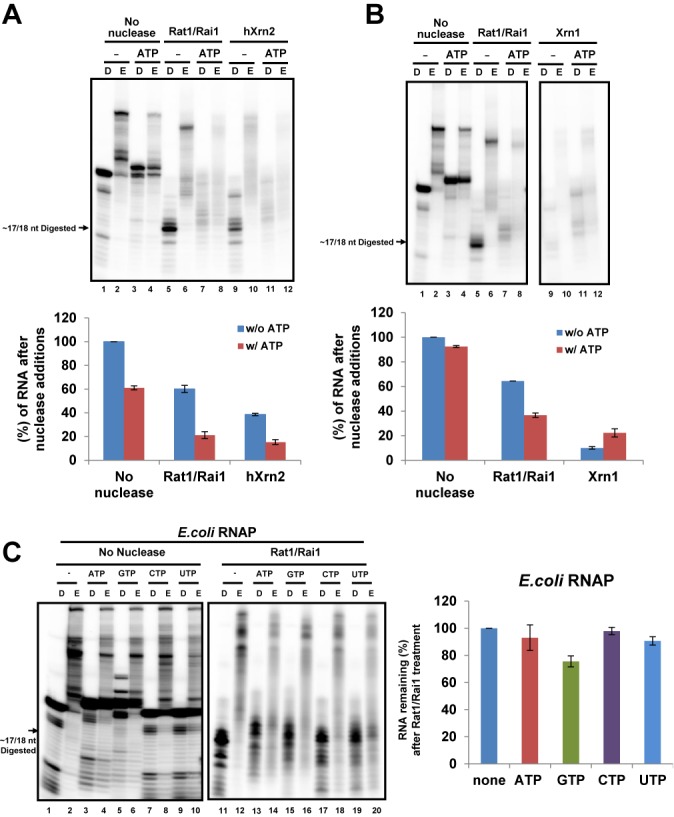
Other 5′-3′ exoribonucleases and *Escherichia coli* RNAP. *In vitro* assays were performed using EC1 scaffold. (**A**) ATP enhances RNAPII termination by recombinant hXrn2 *in vitro*. Quantification of the remaining RNAs is shown below. (**B**) Yeast Xrn1, Rat1's cytoplasmic counterpart, terminates RNAPII regardless of the presence of ATP *in vitro*. Quantification of the remaining RNAs is shown below. (**C**) Rat1/Rai1 cannot terminate *E. coli* RNAP *in vitro* and addition of each NTP had no effect either. Representative gel image is presented on the left and quantification of the remaining RNA is presented in the right panel.

hXrn2 plays a key role in RNAPII termination in humans (5,34), but its terminator function has never been previously investigated *in vitro*. Thus, we examined whether NTP-misincorporation could enhance termination by hXrn2, as observed for Rat1/Rai1. Indeed, hXrn2 more efficiently terminated RNAPII in the presence of ATP (Figure 4A; compare lanes 9 and 11), demonstrating that this mechanism is conserved between yeast and human.

We also tested whether the cytoplasmic 5′-3′ exoribonuclease Xrn1 could mediate RNAPII termination *in vitro*. In contrast to Rat1, Xrn1 terminated RNAPII very effectively even without ATP (Figure 4B, lane 9). This finding was surprising because nuclear-localized Xrn1 was previously shown to be incapable of rescuing the termination defect in a *rat1–1* mutant (35). However, our assay shows that degradation of RNA by the 5′-3′ exonuclease activity of Xrn1 is sufficient to terminate RNAPII, at least *in vitro*. This result implies that Xrn1 may have a higher processivity than Rat1, which would be useful for translocating proteins ahead of it (e.g. RNAPII). Supporting this hypothesis, Xrn1 processively degrades RNA substrates containing stem-loop structures (36,37) and employs an unwinding mechanism via substrate translocation past a steric barrier that excludes double-stranded regions (38). Nuclear-localized Xrn1 may have failed to terminate RNAPII *in vivo* because it cannot interact with RNAPII in a similar fashion as Rat1 and/or other key factors that are necessary for termination.

To investigate whether Rat1 can terminate other polymerases, *E. coli* RNAP was tested. Although Rat1/Rai1 successfully reached *E. coli* RNAP by degrading RNA, it was unable to terminate the polymerase (Figure 4C, lanes 11–20). As previously reported (14), NTP misincorporation occurred in the bacterial RNAP in a similar manner to the yeast RNAPII and reduced the elongation of the polymerase (Figure 4C, lanes 1–10). However, it did not significantly enhance the termination of *E. coli* RNAP by Rat1/Rai1 (Figure 4C, quantification graph). This result suggests that specific protein–protein interaction(s) between Rat1/Rai1 and RNAP may also be critical for triggering termination.

### The length of RNA degraded by Rat1 affects RNAPII termination

**Figure 5.**
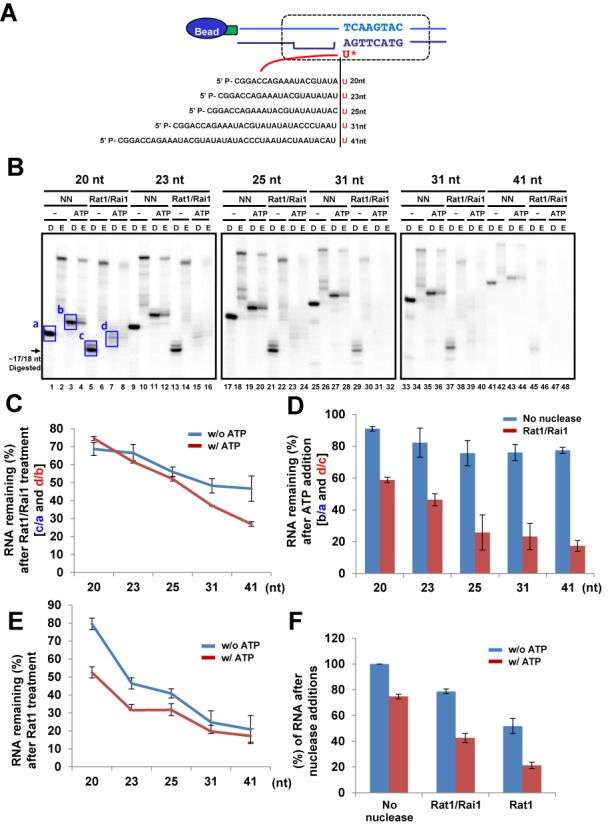
The length of RNA degraded by Rat1 affects RNAPII termination. (**A**) Five ECs harboring different lengths of RNA were tested in the assay. (**B**) Representative gel images of the Rat1/Rai1-treated *in vitro* transcription termination assay. (**C**) Quantification of the remaining RNAs after Rat1/Rai1 treatment without or with ATP. The remaining RNA amounts without or with ATP addition in the no nuclease (NN) groups (a or b, respectively; blue box in gel images) were set to 100% for each EC and the percentage of remaining RNA amounts in the Rat1/Rai1-treated groups without or with ATP treatment (c/a or d/b, respectively) were calculated for each EC. In the presence of ATP, the remaining RNA level after Rat1/Rai1 treatment rapidly decreased as the RNA lengthened. However, this level moderately decreased in the absence of ATP. (**D**) Quantification of the remaining RNAs after ATP addition in no nuclease (NN) or Rat1/Rai1-treated groups. The remaining RNA amounts without ATP addition (a or c, respectively) were set to 100% and the percentage of remaining RNA amounts after ATP addition (b/a or d/c, respectively) were calculated for each EC. The remaining RNA amounts specifically and gradually decreased in the presence of Rat1/Rai1 as the length of the RNA increased. (**E**) Quantification of the remaining RNA amounts without or with ATP addition after Rat1 treatment. There is a dramatic decrease in the remaining RNA level as the RNA length increases but little difference in the remaining RNA levels, regardless of ATP addition, at the ECs harboring 31 and 41 nt RNAs. (**F**) Rat1 terminates RNAPII in a similar fashion to Rat1/Rai1. Addition of ATP also enhances termination by Rat1 alone.

In an attempt to find minimal length of RNA required for RNAPII termination, we tested ECs with variable lengths of RNA (20, 23, 25, 31 and 41 nt) (Figure 5A). We primarily postulated that the length of RNA within the EC might affect the loading of Rat1/Rai1 and subsequent RNAPII termination. The 20 nt RNA was readily degraded by Rat1/Rai1 up to ∼17/18 nt, indicating that an extra 2 or 3 nt are sufficient for recognition by Rat1/Rai1 (Figure 5B; compare lanes 1 and 5). Surprisingly, we found that the termination efficiency increased as the RNA within the EC lengthened (Figure 5B). In the presence of ATP, the remaining RNA level after Rat1/Rai1 treatment drastically decreased from 74.6 (20-nt RNA) to 26.9% (41-nt RNA) (Figure 5C). However, in a control group lacking nuclease, the remaining RNA level was not significantly altered after ATP addition (Figure 5D). We also observed this RNA length effect even in the absence of ATP after Rat1/Rai1 treatment [from 68.7 (20 nt) to 46.7% (41 nt)], although the effect was a little weaker than in the presence of ATP (Figure 5C). These results indicate that RNA degradation step is critical for Rat1/Rai1 to trigger RNAPII termination. One plausible explanation is that Rat1/Rai1 might accumulate a driving force to mechanically dissociate RNAPII from the template while degrading RNAs.

To determine which protein induces the RNA length effect observed, the same experiment was performed without Rai1. Similarly to Rat1/Rai1, Rat1 terminated RNAPII more effectively as it degraded longer RNA (Figure 5E), implying that the RNA length effect is derived from Rat1. However, compared with Rat1/Rai1 (Figure 5C), Rat1 alone terminated RNAPII more efficiently (Figure 5E and F) and achieved the highest level of termination (∼25%) with 41 nt RNA, regardless of ATP addition (Figure 5E). Consistently, hXrn2 also terminated RNAPII better than Rat1/Rai1 (Figure 4A). These results suggest that Rai1 may inhibit or fine-tune the ‘RNAPII-dislodging’ function of Rat1 at the final step, even though it also helps Rat1 to degrade structured RNAs more effectively, which allows Rat1 access to the polymerase (32).

### The 5′-3′ exoribonuclease activity of Rat1 is essential for RNAPII termination

**Figure 6.**
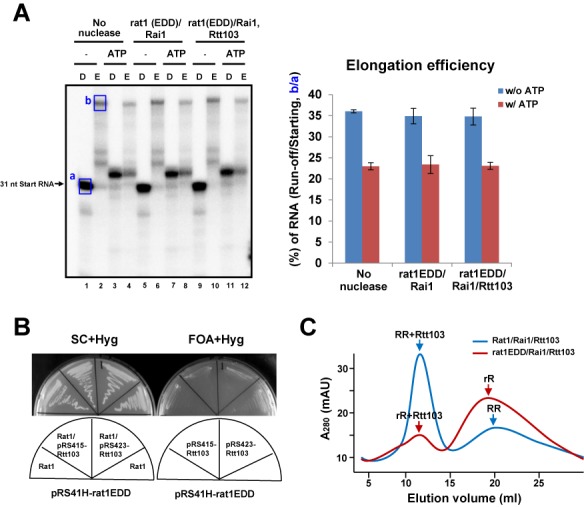
Exoribonucleolytic-deficient rat1 (rat1EDD) cannot terminate RNAPII and Rtt103 does not rescue the termination defect of rat1EDD. (**A**) (Left) rat1EDD/Rai1, regardless of Rtt103 addition, does not terminate RNAPII. (Right) Quantification of the extended run-off RNA amounts relative to the initial starting RNAs without or with ATP addition. rat1EDD does not reduce the RNA elongation efficiency by RNAPII compared with the no nuclease control. (**B**) Multiple copies of the Rtt103 gene cannot rescue the lethality of the rat1EDD mutation. (**C**) Elution profiles of size-exclusion chromatography (Superose 6) of Rat1/Rai1/Rtt103 (RRR) and rat1EDD/Rai1/Rtt103 (rRR) show that most of the Rat1/Rai1 is co-eluted with Rtt103, whereas most of rat1EDD/Rai1 is not. These profiles indicate that rat1EDD shows reduced binding to Rtt103 compared with wild-type Rat1.

To validate the role of 5′-3′ exoribonuclease activity in termination, we generated a catalytically inactive (exo-) form of Rat1 by mutagenizing three conserved acidic residues in the active site (E203A, D233A and D235A). This rat1 mutant (referred to as rat1EDD) was co-expressed and purified as a complex with Rai1 in *E. coli* cells. Because rat1 EDD does not have 5′-3′ exoribonuclease activity, the elongation efficiency (run-off RNAs to initial starting RNAs) rather than the remaining RNA level was measured to determine the extent of RNAPII termination. rat1EDD/Rai1 did not degrade RNAs or decrease RNAPII elongation (Figure 6A, to determine digestion efficiency, compare lanes 5 and 7 with 1 and 3; to determine elongation efficiency, compare the no nuclease group with the rat1EDD/Rai1 group in the quantification graph), confirming that RNA degradation by exonuclease activity is critical to RNAPII termination. A recent study claimed that Rtt103 allows exonucleolytic-deficient Rat1 (D235A) to access and terminate RNAPII, suggesting that the exonucleolytic activity of Rat1 may not be a key feature that triggers termination (9). However, we did not observe a rescue of the termination defect of rat1EDD by adding Rtt103 in the assay (Figure 6A, lanes 9–12). Consistently, the lethality of the rat1EDD mutation was not suppressed by introducing a multi-copy plasmid of the Rtt103 gene (Figure 6B). Furthermore, gel filtration profiles showed that only a small portion of rat1EDD/Rai1 was bound to Rtt103, whereas the majority of wild-type Rat1/Rai1 was complexed with Rtt103 (Figure 6C). This finding indicates that rat1EDD significantly lost its binding affinity for Rtt103, arguing against a role of Rtt103 in bridging exo-rat1 to RNAPII CTD to complement the defective exonuclease activity. The discrepancy could be partially due to additional mutations in the Rat1 active site (D235A versus E203A, D233A, D235A) but is not likely due to the lack of RNAPII CTD serine 2 phosphorylation in our assay system because we detected significant levels of CTD phosphorylation at serine 2 (data not shown). Taken together, our results clearly show that exoribonuclease activity is required for Rat1 not only to approach RNAPII but also to accumulate a sufficient driving force to dislodge the polymerase from the DNA template.

## DISCUSSION

**Figure 7.**
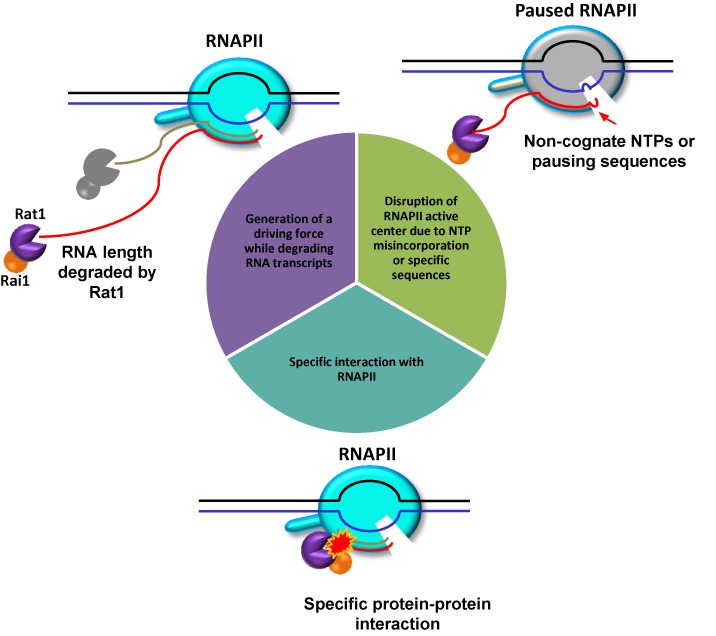
Multiple mechanistic features contribute to Rat1-mediated RNAPII termination. Disruption of the RNAPII active center due to NTP misincorporation or specific sequences facilitated termination by Rat1/Rai1 *in vitro*. A specific interaction between Rat1/Rai1 and RNAPII is critical because Rat1/Rai1 cannot terminate *Escherichia coli* RNAP. Furthermore, Rat1 must degrade RNA transcripts to build up a driving force for termination. Thus, 5′-3′ exonuclease activity is essential for Rat1 not only to gain access to RNAPII but also to accumulate a sufficient driving force to execute termination. Active RNAPII, cyan; paused RNAPII, gray.

In this study, we discovered novel mechanistic features of RNAPII termination by Rat1/Rai1 using an *in vitro* system (Figure 7). First, Rat1/Rai1 more efficiently terminates RNAPII when an NTP misincorporates and induces pausing of the polymerase. Second, Rat1/Rai1 must directly and specifically interact with the target RNAPs to trigger termination, as demonstrated by its inability to terminate *E. coli* RNAP, presumably due to the lack of specific contacts with subunits of bacterial RNAPs. Finally, the length of the RNA degraded is positively correlated with the efficiency of termination. Thus, degradation of RNA by 5′-3′ exoribonuclease activity is not only crucial for Rat1 to gain access to RNAPII, but it may also be important to build up a driving force to dissociate the polymerase.

NTP misincorporation impairs the RNA extension in several ways, including a disruption of the RNAP active site conformation and generation of an offline state of the EC with a frayed RNA 3′-end (23). Paused RNAPII in these states may have subtle changes in the structure near the RNA exit channel and/or in the stability of the DNA–RNA hybrid that can be more easily accessed by Rat1/Rai1. Mismatches often facilitate RNAP backtracking and RNA cleavage, which rescues the elongation function of the paused polymerase. TFIIS stimulates a weak intrinsic cleavage activity of RNAPII (28) and helps to maintain transcriptional fidelity during transcription (21). However, TFIIS occupancy is greatly reduced at the 3′-untranslated region of genes (39), indicating that RNAPII approaching the termination sites might not have TFIIS within the complex which could contribute, at least in part, to the occurrence of mismatches near the termination sites that make RNAPII more vulnerable to Rat1/Rai1.

Although non-cognate NTPs stimulate Rat1-mediated termination, termination efficiencies affected by each non-cognate NTP were not the same (Figure 3), presumably because the extent of misincorporation and mismatch extension apparently differs at each mismatch pair, as demonstrated previously (23). It is also noteworthy that simultaneous addition of Rat1/Rai1 and non-cognate NTPs to the ECs resulted in 20–30% better termination than pre-treatment of non-cognate NTPs to ECs before Rat1/Rat1 addition (data not shown), suggesting that NTPs play another role in Rat1-mediated termination besides misincorporation. As previously hypothesized, co-existing NTPs may activate a cryptic RNase H activity to degrade RNAs within the DNA–RNA hybrid, leading to dissociation of the polymerase (10,32,40). Another interesting study to consider relating to our data is that human TTF2, a Snf/Swi-family factor, dissociates RNAPII from DNA template utilizing the energy from ATP hydrolysis *in vitro* (41,42). It is also recently shown that TTF2-mediated RNAPII termination is tightly regulated by phosphorylation of Gdown1, which predominantly occurs during mitosis (43). Even though we verified that Rat1 does not have an ATPase activity, there is a possibility for Rat1 to utilize ATP in the contact with RNAPII besides misincorporation for better termination.

The bacterial RNAP from *E. coli* was not terminated by Rat1/Rai1 regardless of NTP addition. This result clearly indicates that direct contact between Rat1/Rai1 and the polymerase at the final step of termination should be very specific to dislodge the polymerase. Similarly, another RNAPII termination factor, Sen1, was unable to terminate *E. coli* RNAP (44), indicating that both Rat1 and Sen1 recognize unique features of eukaryotic RNAPII despite the structural similarities between eukaryotic and bacterial polymerases. Rat1 may interact with RNAPII near the RNA exit channel and/or the CTD, but uncovering the precise interaction surface will require structural studies of the Rat1–RNAPII complex.

Termination efficiency is enhanced as the length of RNA degraded by Rat1 increases, suggesting that multiple rounds of processive RNA hydrolysis would lead to more effective termination. We postulate that RNA hydrolysis may allow Rat1 to gradually accumulate a driving force to trigger termination, but the underlying mechanism is currently not understood. Alternatively but not exclusively, Rat1 may require a minimal length of RNA to engage in the termination process. Along the same line, Sen1 was recently shown to require at least an ∼15 nt RNA protruding from RNAPII to elicit termination (44), although the reason is unknown. Because the highly conserved catalytic center of Xrn family proteins accommodates only the 5′-terminal trinucleotides (38), the rest of the nascent RNA transcript may somehow transiently contact Rat1 outside of the active site in this model. This interaction could assist in pulling out the nascent transcript from the RNAPII active center. In either scenario, the RNA length effect manifests an essential role of exonuclease activity in Rat1-mediated termination. Notably, the rat1EDD mutant was unable to terminate RNAPII even when adjacent to the polymerase using the shortest 20 nt RNA (∼3 nt protruding from RNAPII, which allows recognition by Rat1) in the assay (data not shown), which indicates that the RNA degradation process is probably more important to termination than the direct interaction between Rat1 and RNAPII, although we cannot exclude the possibility that the rat1EDD mutant fails to induce anticipated conformational changes of RNAPII as well when it is in contact with the polymerase.

It would be interesting to see how multiple mechanistic features differentially contribute to RNAPII termination when the polymerase is transcribing individual genes. For genes whose transcription termination sites (TTSs) are relatively far from the p(A) sites, degradation of the downstream RNA transcript and subsequent specific RNAPII interaction by Rat1 would be sufficient to trigger termination. However, if the p(A) sites and TTSs are in close proximity, pausing of RNAPII by either NTP misincorporation or pausing specific sequences might help Rat1 to displace the polymerase. However, all these features would be important factors affecting Rat1 termination of transcription at the 3′-end of genes.

## SUPPLEMENTARY DATA

Supplementary Data are available at NAR Online.

SUPPLEMENTARY DATA
