# Supplementary material for: Unraveling the mechanistic features of RNA polymerase II termination by the 5′-3′ exoribonuclease Rat1
Source: Nucleic Acids Res. 2015 Feb 26;43(5):2625–37. doi: 10.1093/nar/gkv133 (PMC4357727; doi:10.1093/nar/gkv133)
Supplement: SUPPLEMENTARY DATA [file supp_43_5_2625__index.html]

Unraveling the mechanistic features of RNA polymerase II termination by the 5′-3′ exoribonuclease Rat1 — SUPPLEMENTARY DATA 

# Unraveling the mechanistic features of RNA polymerase II termination by the 5′-3′ exoribonuclease Rat1

## SUPPLEMENTARY DATA

**Files in this Data Supplement:**

- Supplementary Table 1
