# Supplementary material for: Unraveling the mechanistic features of RNA polymerase II termination by the 5′-3′ exoribonuclease Rat1
Source: Nucleic Acids Res. 2015 Feb 26;43(5):2625–37. doi: 10.1093/nar/gkv133 (PMC4357727; doi:10.1093/nar/gkv133)
Supplement: SUPPLEMENTARY DATA [file supp_gkv133_nar-03135-v-2014-File003.docx]

**SUPPLEMENTARY TABLE 1.**

**RNA and DNA oligonucleotides sequence**

| **Oligonucleotide** | **Sequences** |
| --- | --- |
| EC1 Template DNA | 5’-CCAGTCATCGTCGACTGACTCCAAGCTCAAGTACTTGAGCC  TGGTCATTACTAGTACTGCCTTGACCTAGCGTCGG-3’ |
| EC1 Non-Template DNA | 5’BIOTIN-GGCTACCGACGCTAGGTCAAGGCAGTACTAGTAATG  ACCAGGCTCAAGTACTTGAGCTTGGAGTCAGTCGACGATGACTGG-3’ |
| EC2 Template DNA | 5’-CCAGTCATCGTCGACTGACTCCAAGCTCAAGTACTGGAGCC  TGGTCATTACTAGTACTGCCTTGACCTAGCGTCGG -3’ |
| EC2 Non-Template DNA | 5’BIOTIN- GCTACCGACGCTAGGTCAAGGCAGTACTAGTAATG  ACCAGGCTCCAGTACTTGAGCTTGGAGTCAGTCGACGATGACTGG -3’ |
| RNA 19 | 5’P-AUAUGCAUAAAGACCAGGC -3’ |
| RNA 22 | 5’P-UAUAUAUGCAUAAAGACCAGGC -3’ |
| RNA 24 | 5’P-CAUAUAUAUGCAUAAAGACCAGGC -3’ |
| RNA 30 | 5’P-UAAUCCCAUAUAUAUGCAUAAAGACCAGGC -3’ |
| RNA 40 | 5’P-UACAUAAUCAUAAUCCCAUAUAUAUGCAUAAAGACCAG  GC -3’ |
